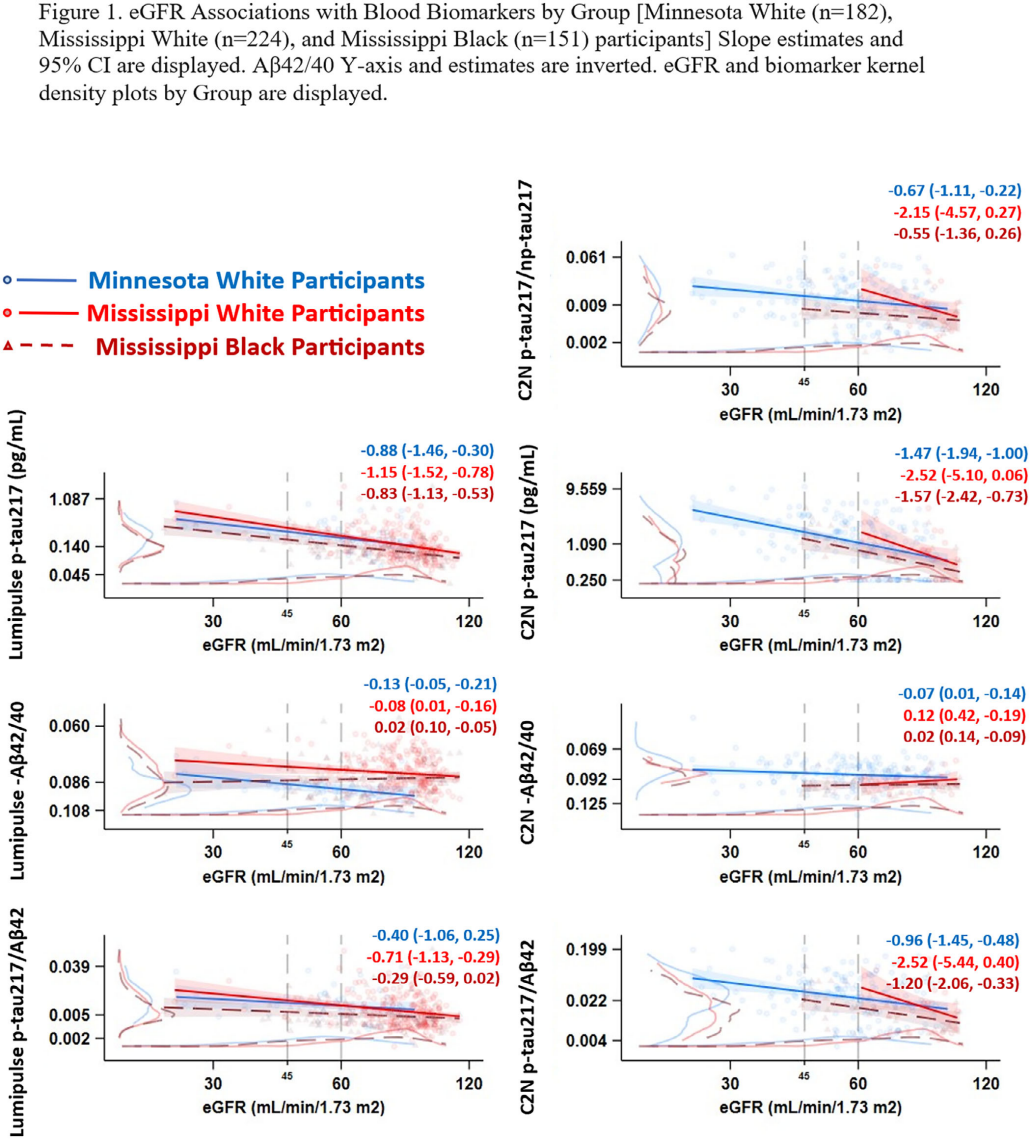# Plasma Alzheimer's Disease Biomarker Associations with eGFR in Black and White Older Adults

**DOI:** 10.1002/alz70856_107141

**Published:** 2026-01-08

**Authors:** Kevin J. Sullivan, Chad T Blackshear, Joshua A Bornhorst, David S. Knopman, Jeremy A. Syrjanen, Maria Vassilaki, Daniel Figdore, Clifford R. Jack, Ronald Petersen, Gwen Gwen Windham, Thomas H. Mosley, Alicia Algeciras‐Schimnich, Michael E. Griswold

**Affiliations:** ^1^ University of Mississippi Medical Center, The MIND Center, Jackson, MS, USA; ^2^ Mayo Clinic, Rochester, MN, USA; ^3^ Department of Radiology, Mayo Clinic, Rochester, MN, USA; ^4^ Mayo Clinic Alzheimer's Disease Research Center, Rochester, MN, USA; ^5^ Department of Neurology, Mayo Clinic, Rochester, MN, USA

## Abstract

**Background:**

Decreased kidney function, indicated by a lower estimated glomerular filtration rate (eGFR), may affect interpretation of Alzheimer's disease (AD) blood biomarkers, including phosphorylated tau (*p*‐tau) and amyloid‐beta (Aβ) peptides, due to altered clearance. However, there is limited data available to determine the influence of eGFR on these biomarkers and whether it differs by biomarker or assay type, especially in racially diverse studies of community‐based older adults.

**Methods:**

We used data from 557 participants in the Mayo Clinic Study of Aging (Rochester, MN *n* = 182; age 79±8 years; 36.3% Female, White participants) and the UMMC MIND Center‐Mayo Clinic Study of Aging (Jackson, MS *n* = 375; age 69±8 years; 73% Female 27% Black participants). eGFR was determined using the 2021 CKD‐EPI Creatinine equation. Plasma biomarkers were measured using the Lumipulse immunoassays for *p*‐tau217, Aβ42, and Aβ40 (Fujirebio Diagnostics; *n* = 420); or the mass spectrometry‐based PrecivityAD2 assay (C2N Diagnostics; *n* = 253) for *p*‐tau217, np‐tau217, Aβ42, and Aβ40. Common ratio values were calculated. Aβ42/40 measures were inverted so higher values are worse for all biomarkers. Log base‐2 transformed regression analysis related eGFR to each single analyte and ratio; coefficients (95% CI) reflect estimated doubling in biomarker per doubling in eGFR. Group specific [MN White (*n* = 182), MS White (*n* = 224), MS Black (*n* = 151) participants] estimates are reported, as the distribution of social determinants of health can vary across populations and locations.

**Results:**

Lower eGFR was associated with higher (worse) AD‐plasma biomarkers, and group stratified estimates were mostly consistent (Figure 1). Ratios of biomarkers diminished but did not entirely alleviate associations with eGFR. For example, MN White participants’ C2N *p*‐tau217 association of ‐1.47 (‐1.94, ‐1.00), was attenuated to ‐0.67 (‐1.11, ‐0.22), when using *p*‐tau217/np‐tau217.

**Conclusions:**

Kidney function may be associated with AD‐plasma biomarker values, even with common ratio corrections. There was not strong evidence for highly differential associations of eGFR by race/site categories, though sample sizes are currently limited. The implications for diagnosis and risk classification need to be explored further to support health equity. Optimal consideration of comorbidities on blood biomarker concentrations must continue to be informed by investigation in large, racially and ethnically diverse, community‐based studies.